# Standard Thermodynamic Properties, Biosynthesis Rates, and the Driving Force of Growth of Five Agricultural Plants

**DOI:** 10.3389/fpls.2021.671868

**Published:** 2021-05-31

**Authors:** Marko Popovic, Mirjana Minceva

**Affiliations:** Biothermodynamics, TUM School of Life Sciences, Technical University of Munich, Freising, Germany

**Keywords:** bean (*Phaseolus vulgaris* L.), rice (*Oryza sativa* L.), *Gossypium* (cotton), Sugarcane (*Saccharum* spp.), corn (*Zea mays* L.), phototroph, biothermodynamics, Gibbs energy

## Abstract

Elemental composition of *Gossypium hirsutum* L. (cotton), *Oryza sativa* L. (Asian rice), *Phaseolus vulgaris* L. (common bean), *Saccharum* spp. L. (sugarcane), and *Zea mays* L. (corn) was used to calculate their empirical formulas (unit carbon formulas) and growth stoichiometry. The empirical formulas were used to find standard enthalpy of formation, standard molar entropy, standard Gibbs energy of formation, and standard molar heat capacity. A comparison was made between thermodynamic properties of live matter of the analyzed plants and other unicellular and multicellular organisms. Moreover, the growth process was analyzed through standard enthalpy, entropy, and Gibbs energy of biosynthesis. The average standard Gibbs energy of biosynthesis was found to be +463.0 kJ/C-mol. Thus, photosynthesis provides energy and carbon for plant growth. The average intercepted photosynthetic energy was found to be 15.5 MJ/C-mol for the analyzed plants. However, due to inefficiency, a great fraction of the intercepted photosynthetic energy cannot be used by plants. The average usable photosynthetic energy was found to be –2.3 MJ/C-mol. The average thermodynamic driving force for growth is –1.9 MJ/C-mol. Driving forces of growth of C3 and C4 plants were compared. It was found that C4 plants have a greater driving force of growth than C3 plants, which reflects the greater efficiency of C4 photosynthesis. The relationship between the driving force and growth rates was analyzed by determining phenomenological *L* coefficients. The determined phenomenological coefficients span two orders of magnitude, depending on plant species and environmental conditions. The *L* coefficient of *P. vulgaris* was found to be lower than that of other plants, due to additional energy requirements of nitrogen fixation.

## Graphical Abstract

Intercepted photosynthetic energy *q* is partly converted into usable photosynthetic energy, Δ*_*ps*_G*, with an efficiency *μ*. A part of Δ*_*ps*_G* is used for biosynthesis Δ*_*bs*_G*, which is represented by the growth reaction in the bottom, where “Bio” denotes newly formed live matter. The remaining energy is the driving force of growth, Δ*_*DF*_G*, used to make growth at a desired rate. The background for the figure was taken from Pixabay (https://www.pixabay.com/), image ID number: 4779065, under Pixabay license.

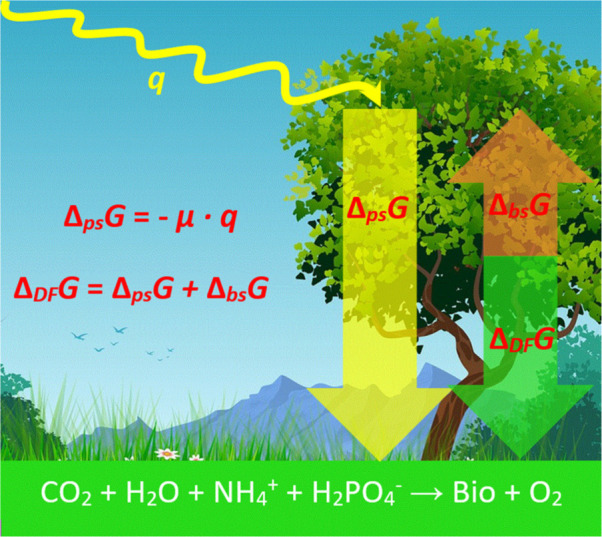

## Introduction

Thermodynamics has a great potential in life sciences (Von Stockar and Liu, 1999; Von Stockar, 2010, 2013a; Lucia, 2015). Thermodynamics has been used in systems biology to analyze metabolic pathways and analyze metabolic strategies evolved by microorganisms (Von Stockar and Liu, 1999; Von Stockar, 2013b). Thermodynamics is used more and more often in biology for analysis of microorganism cells and communities (Hellingwerf et al., 1982; McInerney and Beaty, 1988; Del Giorgio and Cole, 1998; Kleerebezem and Van Loosdrecht, 2010; Soh and Hatzimanikatis, 2010), as well as plants (Dragicevic and Sredojevic, 2011; Keller, 2013). Morowitz (1968) was the first to roughly estimate thermodynamic properties of ubiquitously present microorganisms using statistical thermodynamics. Afterward, the interest in biothermodynamics increased, which motivated researchers to study the subject in more detail. Wang et al. (1976) and Duboc et al. (1999) reported experimental enthalpies of combustion of microorganisms. The next great advance in the field was made by Battley et al. (1997) by experimentally determining the entropy of *Saccharomyces cerevisiae*. Based on this data, Battley (1998) has made a very detailed analysis of *S. cerevisiae* growth, determining the change in specific enthalpy, entropy, and Gibbs free energy for the process in both aerobic and anaerobic modes and on several substrates. Barros and Feijóo (2003) made a quantitative analysis of soil microorganism growth, describing it with a growth reaction. Multicellular and unicellular organisms represent a highly organized amount of substance, containing nucleic acid, proteins, lipids, and carbohydrates, characterized by specific elemental composition (empirical formula) and thermodynamic properties (enthalpy, entropy, and Gibbs energy) (Von Stockar, 2013a; Ozilgen and Sorguven, 2016). The conversion of energy by plants must be performed in full accordance with the general laws of thermodynamics (Ksenzhek and Volkov, 1998). Plant growth rate is proportional to the product of the metabolic rate and the metabolic efficiency for production of anabolic products (Criddle et al., 2005).

Thermodynamics has been used to study plant growth and photosynthesis. Hansen et al. (1994) related metabolic heat and CO_2_ rates with the plant growth rate and carbon-conversion efficiency, by developing a model based on physiological parameters. Criddle et al. (2005) found that plants have evolved to have maximum growth rates at temperatures most common in their environment. Moreover, analysis of the energetics of respiration shows that measurements of metabolic heat and CO_2_ rates by calorespirometry, combined with estimates of substrate and biomass composition, are sufficient to calculate substrate carbon-conversion efficiencies, anabolic rates or rates of growth and development, and relative activities of metabolic paths (Macfarlane et al., 2002; Hansen et al., 2005). Dragicevic and Sredojevic (2011) used thermodynamic principles to analyze plant physiological processes, including seed germination and plant growth. Keller (2013) analyzed the energetics of photosynthesis. Enquist et al. (1998, 1999) found that energetic constraints on metabolism are reflected in population density, and other ecological and evolutionary phenomena, including resource allocation among species in ecosystems. Attorre et al. (2019) developed a model of plant growth based on non-equilibrium thermodynamics, which was able to predict CO_2_ fixation by plants, as well as their response to changes in their environment. Colombi et al. (2019) used isothermal calorimetry to study energy used for root growth and penetration through the soil. Herrmann and Colombi (2019) used calorespirometry to study energy use efficiency of root growth. Ksenzhek and Volkov (1998) analyzed the applications of thermodynamic principles to plant growth and metabolism.

Growth of organisms requires a thermodynamic driving force, in the form of negative Gibbs energy (Von Stockar, 2013b). While the value of the driving force of higher plants remains unknown, it has been determined for heterotrophic microorganisms (Von Stockar and Liu, 1999; Von Stockar, 2013b) and microalgae. Von Stockar et al. (2011) used calorimetry to measure thermodynamic properties of photosynthetic growth of the microalgae *Chlorella vulgaris*, strain CCAP 211/11B. They found that the culture absorbed –5,000 kJ of light per C-mol of biomass formed (Von Stockar et al., 2011). This energy is partly used to cover the cost of biosynthesis of new biomass, which was measured to be +458 kJ/C-mol (Von Stockar et al., 2011). The remaining –4,500 kJ/C-mol represents the driving force for growth (Von Stockar et al., 2011). This excess driving force is required to keep the reaction sufficiently far from equilibrium for it to occur at a desired rate, since the reaction rate is proportional to distance from equilibrium (Hellingwerf et al., 1982; Westerhoff et al., 1982; Balmer, 2011; Von Stockar, 2013b,c; Demirel, 2014; Popovic and Minceva, 2020a,b).

Solar radiation is the ultimate source of energy for most life, with the exceptions of hydrothermal vents in the deep ocean. It is harvested by autotrophs and then distributed through food webs in the form of chemical energy. The role of photosynthesis in plant growth has been extensively analyzed in the literature (Pearcy et al., 1981; Allen et al., 1988; Kirschbaum, 2011; Holding and Streich, 2013; Weraduwage et al., 2016). However, the absorbed light energy goes through intermediate steps before it can be used to drive metabolic processes. It is first converted into chemical energy in the form of photosynthate, which is then partly oxidized in catabolism to produce ATP for metabolic processes (e.g., maintaining homeostasis and growth). A detailed discussion of thermodynamics of plant catabolism and respiration is given in Hansen et al. (1994, 2004, 2021), Gary et al. (1995), and Ellingson et al. (2003).

The goal of this research is to make a thermodynamic analysis of growth of five widely cultivated plants: *Gossypium hirsutum* L. (cotton), *Oryza sativa* L. (Asian rice), *Phaseolus vulgaris* L. (common bean), *Saccharum* spp. L. (sugarcane), and *Zea mays* L. (corn). Elemental compositions of the analyzed plants will be used to calculate empirical formulas, thermodynamic properties of plant tissues, and their growth, as well as to formulate growth reactions. After that, the calculated thermodynamic properties will be compared with those of viruses, bacteria, fungi, archaea, and human tissues. This will show whether there are significant differences in the driving force of growth of phototrophic and chemotrophic organisms, as well as between prokaryotes and eukaryotes. Moreover, the driving force will be related to the growth rate using nonequilibrium thermodynamics.

## Materials and Methods

Like all other organisms, plants have characteristic empirical formulas and thermodynamic properties of live matter. Their determination, starting from elemental composition data, is discussed in the *Properties of Live Matter* section. The empirical formulas are then used to find stoichiometry of growth and thermodynamic properties of biosynthesis, as discussed in the *Properties of Growth and Biosynthesis* section. The determined properties have an uncertainty, as discussed in the *Uncertainties* section.

### Properties of Live Matter

The starting point for this research is elemental composition of plant dry matter reported in the literature. Elemental composition of live matter is measured as mass fractions, which are then converted into unit carbon formulas, also known as C-mol or empirical formulas (Battley, 2013). The general unit carbon formula of the analyzed plants is C_*nC*_H_*nH*_O_*nO*_N_*nN*_P_*nP*_S_*nS*_Na_*nNa*_K_*nK*_Mg_*nMg*_Ca_*nCa*_Cl_*nCl*_, where *n*_*J*_ is the atomic coefficient of element J, which can be calculated using the equation

(1) $$n_{J}=\frac{w_{J}}{w_{C}}\frac{M_{C}}{M_{J}}$$

where *w*_*J*_ and *w*_*C*_ are the mass fractions of element J and carbon in the biomass, respectively, while *M*_*J*_ and *M*_*C*_ are the molar masses of element J and C, respectively (Duboc et al., 1999). Empirical formulas are reported for organism dry matter (excluding water) (Duboc et al., 1999). Molar masses of empirical formulas, *M*_*bio*_, can be found through the equation (Duboc et al., 1999)

(2) $$M_{bio}=\frac{1}{w_{C}}M_{C}$$

Thermodynamic properties of live matter were calculated as described in, Patel and Erickson (1981); Hurst and Harrison (1992), Battley (1998, 1999), and Popovic (2019). Elemental composition of animate matter can be used to determine its enthalpy of formation through the Patel–Erickson equation and classical reaction thermochemistry. The Patel–Erickson equation relates heat released during combustion to number of electrons transferred to oxygen (Patel and Erickson, 1981; Battley, 1998):

(3) $${△}_{C}H^{0}\left(bio\right)=-111.14\frac{\text{kJ}}{\text{mol}}\cdot E$$

where Δ*_*C*_H*^0^ is standard enthalpy of combustion of live matter and *E* is the number of electrons transferred to oxygen during combustion to CO_2_(g), H_2_O(l), N_2_(g), P_4_O_1__0_(s), and SO_3_(g) (for a discussion on other conventions concerning SO_3_ please see Popovic, 2019). During combustion, a C atom gives its four valence electrons to O, H gives one, N gives none since it is converted to N_2_, P gives five, and S gives six. Inorganic ions, like Na^+^ and Mg^2+^, are not included, since they are already in their highest oxidation state and cannot transfer any electrons to oxygen (Battley, 1998). Thus, *E* is calculated through the equation:

(4) $$E=4n_{C}+n_{H}-2n_{O}-0n_{N}+5n_{P}+6n_{S}\;$$

where *n*_*C*_, *n*_*H*_, *n*_*O*_, *n*_*N*_, *n*_*P*_, and *n*_*S*_ are, respectively, the number of C, H, O, N, P, and S atoms in the biomass empirical formula (Patel and Erickson, 1981; Battley, 1998). If any of these atoms are not present in the empirical formula, they are just neglected during the calculation (Battley, 1998). Standard enthalpy of combustion of live matter Δ*_*C*_H*^0^(*bio*) is the standard enthalpy change of the reaction.

(5) $$\begin{array}{l} C_{nC}H_{nH}O_{nO}N_{nN}P_{nP}S_{nS}K_{nK}Mg_{nMg}Ca_{nCa}Al_{nAl}Si_{nSi}Mn_{nMn}Fe_{nFe}\\ Cl_{nCl}+(n_{C}+¼n_{H}+1¼n_{P}+1½n_{S}+¼n_{K}+½n_{Mg}\\ +½n_{Ca}+¾n_{Al}+n_{Si}+n_{Mn}+¾n_{Fe}-½n_{O}-¼n_{Cl})O_{2}\;\to \\ n_{C}CO_{2}+½n_{H}H_{2}O+½n_{N}N_{2}+¼n_{P}P_{4}O_{10}+n_{S}SO_{3}+½n_{K}\\ K_{2}O+n_{Mg}MgO+n_{Ca}CaO+½n_{Al}Al_{2}O_{3}+n_{Si}SiO_{2}+n_{Mn}\\ MnO_{2}+½n_{Fe}Fe_{2}O_{3}+n_{Cl}HCl \end{array}$$

Thus, Δ*_*C*_H*^0^ can be used to find standard enthalpy of formation, Δ*_*f*_H*^0^(*bio*), of live matter, through the Hess law (Battley, 1998; Popovic, 2019).

(6) $$\begin{array}{l} {△}_{f}H^{0}(bio)=n_{C}{△}_{f}H^{0}(CO_{2})+\frac{1}{2}n_{H}{△}_{f}H^{0}(H_{2}O)+\frac{1}{4}n_{P}{△}_{f}H^{0}\\ \left(P_{4}O_{10})+n_{S}{△}_{f}H^{0}(SO_{3})+\frac{1}{2}n_{K}{△}_{f}H^{0}(K_{2}O)+n_{Mg}{△}_{f}H^{0}(MgO\right)\\ +n_{Ca}{△}_{f}H^{0}(CaO)+\frac{1}{2}n_{Al}{△}_{f}H^{0}(Al_{2}O_{3})+n_{Si}{△}_{f}H^{0}(SiO_{2})\\ +n_{Mn}{△}_{f}H^{0}(MnO_{2})+\frac{1}{2}n_{Fe}{△}_{f}H^{0}(Fe_{2}O_{3})+n_{Cl}{△}_{f}H^{0}(HCl)\\ -{△}_{C}H^{0}(bio) \end{array}$$

Elemental composition can also be used to determine standard *molar* entropy of live matter, *S*^0^*_*m*_*(*bio*), through the Battley equation (Battley, 1999)

(7) $$S_{m}^{o}\left(bio\right)=0.187\sum_{J}\frac{S_{m}^{o}\left(J\right)}{a_{J}}n_{J}$$

where *n*_*J*_ is the number of atoms of element *J* in the empirical formula of the biomass, *S*^0^*_*m*_*(*J*) is standard molar entropy of element *J*, and *a*_*J*_ is the number of atoms per molecule of element *J* in its standard state elemental form. For example, the standard state elemental form of carbon is graphite, which is simply written as C, which makes *a*_*C*_ = 1. On the other hand, hydrogen, oxygen, and nitrogen are in their standard state elemental forms of all diatomic gasses H_2_, O_2_, and N_2_, respectively, which implies that *a*_*H*_ = *a*_*O*_ = *a*_*N*_ = 2. The summation is over all elements constituting the dry live matter. The Battley equation can also be used to determine standard entropy *of formation* of live matter Δ*_*f*_S*^0^(*bio*). In this case, it takes the form (Battley, 1999)

(8) $${△}_{f}S^{0}\left(bio\right)=-0.813\sum_{J}\frac{S_{m}^{o}\left(J\right)}{a_{J}}n_{J}$$

Finally, by combining standard enthalpy and entropy of formation, it is possible to calculate standard Gibbs energy of formation of live matter Δ*_*f*_G*^0^(*bio*), as

(9) $${△}_{f}G^{0}\left(bio\right)={△}_{f}H^{0}\left(bio\right)-T{△}_{f}S^{0}\left(bio\right)$$

where *T* is the temperature.

Standard molar heat capacities of the analyzed plants were determined using the Hurst–Harrison equation. The Hurst–Harrison equation is a group contribution model, describing standard molar heat capacity of a substance, *C*^0^*_*p*_*,*_*m*_*, as a sum of contributions from its constituent elements

(10) $$C_{p,m}^{o}=\sum_{J}n_{J}c_{J}$$

where *n*_*J*_ is the number of atoms of element *J* in the substance formula, while *c*_*J*_ is an empirical parameter that describes the contribution of element *J* to heat capacity (Hurst and Harrison, 1992; Ozilgen and Sorguven, 2016). The values of *c*_*J*_ can be found in Hurst and Harrison (1992).

### Properties of Growth and Biosynthesis

Growth is a chemical process in which an organism converts nutrients into new live matter, a process that can be represented by chemical reactions, known as growth reactions (Raven, 1985; Battley, 1998, 2013; Von Stockar, 2013b; Pineda-Insuasti et al., 2014; Popovic and Minceva, 2020a). Growth reactions describe the stoichiometry with which nutrients are converted into live matter.

The non-metals C, H, N, O, P, and S, also known as CHNOPS elements, are essential for all living systems, forming the majority of cell structures (Wackett et al., 2004). Carbon enters photosynthetic tissues of plants as carbon dioxide. Hydrogen and oxygen enter plants mostly through the root as water. Nitrogen enters plants mainly through roots in the form of ammonium or nitrate salts. Phosphorus enters plants predominantly from soil in the form of phosphate salts. Sulfur is taken up by plants mostly from soil in the sulfate form (Linzon et al., 1979). Except through soil, plants can take many elements through leaves from foliar fertilizers, which can reduce the total amount of fertilizer applied and achieve high fertilizer efficiency (Niu et al., 2021).

Except for the CHNOPS elements, plants use a great number of metallic ions, which are taken predominantly from the soil through the roots. Potassium greatly influences plant growth and is taken as K^+^ (Ragel et al., 2019; Xu et al., 2020). Magnesium is important in protein synthesis and is imported by plants in the form of Mg^2+^ (Verbruggen and Hermans, 2013; Guo et al., 2016; Hauer-Jákli and Tränkner, 2019). Calcium is taken as Ca^2+^ (White et al., 2002; White and Broadley, 2003) and has a dual function as a structural component of cell walls (Hepler, 2005; Thor, 2019) and membranes and as intracellular second messenger (Knight et al., 1997; Blume et al., 2000; Michard et al., 2011; Monshausen et al., 2011; Wilkins et al., 2016; Ortiz-Ramírez et al., 2017; Tang and Luan, 2017; Kudla et al., 2018; Thor, 2019). Silicon is taken in the form of silicic acid Si(OH)_4_ (Ma and Yamaji, 2006) and has beneficial effects on growth, development, yield, and disease resistance of many plant species (Ma, 2004; Ma and Yamaji, 2006; Luyckx et al., 2017). Aluminum is present in plants as Al^3+^ ion (Bojórquez-Quintal et al., 2017). In small amounts, aluminum stimulates root growth, increases nutrient uptake, and improves enzyme activity but inhibits root growth at higher concentrations (Bojórquez-Quintal et al., 2017). Manganese is taken as Mn^2+^ and is an important cofactor for catalyzing the water-splitting reaction in photosystem II (Alejandro et al., 2020). Iron is present in the soil mostly as Fe^3+^ ions but can also be present as Fe^2+^ in smaller amounts. Iron is imported by plants using various strategies (Morrissey and Guerinot, 2009; Jacoby et al., 2017; Mhlongo et al., 2018) and is used in many processes, including electron transfer chains and photosynthesis (Schmidt et al., 2020). Chlorine is taken as Cl^–^ and is important in osmoregulation, stomata opening, and photosynthesis (Critchley, 1985; Coleman et al., 1987; Chen et al., 2010; Marschner, 2011; Colmenero-Flores et al., 2019).

Other elements, such as Cu and Zn, are also used by plants. However, the literature did not contain sufficient data to include these elements into the calculations. Since their amount is relatively small compared with the CHNOPS elements (Wackett et al., 2004), they do not influence thermodynamic properties of plants. However, it would be interesting to consider their influence on plant nutrition in the future from the perspective of biothermodynamics and growth reactions.

Based on the discussion above, the general growth reaction proposed for the analyzed plants is

(11) $$\begin{array}{l} CO_{2}(g)+H_{2}O(l)+NH_{4}{}^{+}(aq)+H_{2}PO_{4}{}^{-}(aq)+SO_{4}{}^{2-}\\ \left(aq)+K^{+}(aq)+Mg^{2+}(aq)+Ca^{2+}(aq)+Al^{3+}(aq\right)\\ +Si(OH){\;}_{4}(S)+Mn^{2+}(aq)+Fe^{3+}(aq)+Cl^{-}(aq)\to \\ Bio+O_{2}(g)+H^{+}(aq) \end{array}$$

where “Bio” denotes live matter (excluding water). Stoichiometric coefficients of nutrients and metabolic products are specific for each plant species and are discussed in the *Live Matter Elemental Composition and Stoichiometry of Growth* section.

Growth reaction thermodynamic parameters were calculated using classical thermochemistry

(12) $${△}_{bs}H^{0}=\sum_{products}\nu {△}_{f}H^{0}-\sum_{reactants}\nu {△}_{f}H^{0}$$

(13) $${△}_{bs}S^{0}=\sum_{products}\nu S_{m}^{o}-\sum_{reactants}\nu \;S_{m}^{o}$$

(14) $${△}_{bs}G^{0}=\sum_{products}\nu {△}_{f}G^{0}-\sum_{reactants}\nu {△}_{f}G^{0}$$

where *ν*’s are stoichiometric coefficients of species participating in the reaction, while Δ*_*bs*_H*^0^, Δ*_*bs*_S*^0^, and Δ*_*bs*_G*^0^ are standard enthalpy, entropy, and Gibbs energy of biosynthesis, respectively (Atkins and de Paula, 2011; Atkins et al., 2017).

### Uncertainties

Thermodynamic properties of the studied plants were determined from elemental composition using empirical relations and thus have some uncertainty. Δ*_*C*_H*^0^ was found using the Patel–Erickson equation, the uncertainty of which is 5.36% (Popovic, 2019). The determined Δ*_*C*_H*^0^ values were then subtracted from standard enthalpies of formation of oxides (Equation 6) to find Δ*_*f*_H*^0^(*bio*). Since standard enthalpies of formation of oxides were precisely determined by experiment (more details in Chase, 1998), they have a negligible error compared with that in Δ*_*C*_H*^0^. Thus, the uncertainty in standard enthalpy of formation of live matter, δ(Δ*_*f*_H*^0^(*bio*)), is equal to the error in Δ*_*C*_H*^0^.

(15) $$\delta \left({△}_{f}H^{0}\left(bio\right)\right)=0.0536\cdot \left[111.14\frac{kJ}{mol}\left(4n_{C}+n_{H}-2n_{O}-0n_{N}+5n_{P}+6n_{S}\right)\right]$$

The uncertainty in estimation of entropy using the Battley equation is 2% for dry matter and 19.7% for hydrated matter (Battley, 1999). The greater of the two values was taken, and hence the uncertainty in standard molar entropy of live matter, δ(*S*^0^*_*m*_*(*bio*)), is

(16) $$\delta \left(S_{m}^{0}\left(bio\right)\right)=0.197\cdot S_{m}^{0}\left(bio\right)$$

Δ*_*f*_S*^0^(*bio*) is defined as the standard entropy change of the reaction of formation of live matter from its constituent elements in their standard forms.

(17) $$\begin{array}{l} n_{c}C(\text{s},graphite)+½n_{H}H_{2}(g)+½n_{O}O_{2}(g)+½n_{N}N_{2}(g)\\ +\;n_{p}P(\text{s},white)+n_{s}S(\text{s},rhombic)+n_{Na}Na(s)+n_{K}K(s)\\ +n_{Mg}Mg(s)+n_{Ca}Ca(s)+½n_{Cl}Cl_{2}(g)\to C_{nC}H_{nH}O_{nO}\\ N_{nN}P_{nP}S_{nS}Na_{nNa}K_{nK}Mg_{nMg}Ca_{nCa}Cl_{nCl} \end{array}$$

and is defined as the difference in *S*^0^*_*m*_*(*bio*) and standard molar entropies of the elements, which have been determined with great accuracy by experiment (Chase, 1998). Thus, the uncertainty in Δ*_*f*_S*^0^(*bio*) is equal to that in *S*^0^*_*m*_*(*bio*) (Popovic, 2019).

Δ*_*f*_H*^0^(*bio*) and Δ*_*f*_S*^0^(*bio*) are used to find Δ*_*f*_G*^0^(*bio*). Therefore, the uncertainty in the standard Gibbs energy of formation of live matter, δ(Δ*_*f*_G*^0^(*bio*)), is

(18) $$\delta \left({△}_{f}G^{0}\left(bio\right)\right)=\sqrt{{\left[\delta \left({△}_{f}H^{0}\left(bio\right)\right)\right]}^{2}+{\left[T\cdot \delta \left(S_{m}^{0}\left(bio\right)\right)\right]}^{2}}$$

Finally, the uncertainty in Δ*_*f*_G*^0^(*bio*) is equal to that in Δ*_*bs*_G*^0^, since it is the greatest source of uncertainty in its determination. Δ*_*bs*_G*^0^ is determined using Equation (14), as the difference of Δ*_*f*_G*^0^ values of reactants and products of reaction (Equation 11). The Δ*_*f*_G*^0^ values of all reaction participants, except for live matter, have been determined with great accuracy by experiment (Chase, 1998). Thus, uncertainty in Gibbs energy of biosynthesis, δ(Δ*_*bs*_G*^0^), is equal to δ(Δ*_*f*_G*^0^(*bio*)). Similarly, δ(Δ*_*bs*_H*^0^) and δ(Δ*_*bs*_S*^0^) are equal to δ(Δ*_*f*_H*^0^(*bio*)) and *S*^0^*_*m*_*(*bio*), respectively.

## Results and Discussion

Elemental composition and thermodynamic properties of plants and their growth are discussed in the following sections.

### Live Matter Elemental Composition and Stoichiometry of Growth

Elemental compositions of the five plant species were taken from the literature (Latshaw and Miller, 1924; Medic et al., 2012; Titiloye et al., 2013; Pineda-Insuasti et al., 2014; Nam and Capareda, 2015; Fialho et al., 2019). Information about the plant samples is summarized in Table 1, including geographic origin, plant parts, and whether experimental enthalpy of combustion was reported. Calorimetrically determined enthalpies of combustion were reported for *Gossypium hirsutum* L., *Oryza sativa* L., *Saccharum* spp. L., and *Zea mays* L. stalk and cobs from Ghana (Titiloye et al., 2013; Nam and Capareda, 2015). Since for these samples Δ*_*C*_H*^0^(*bio*) was determined directly by experiment instead of Equation (4), their standard enthalpies of formation, Δ*_*f*_H*^0^(*bio*), are more accurate. Elemental compositions of all the analyzed plants have been reported for dry matter.

**TABLE 1:** Information about the plant samples considered in this research.

Plant name		Sample origin	Plant part	Experimental enthalpy of combustion	References
Latin	Common				
*Gossypium hirsutum* L.	Cotton	California	Stalk	Yes	Nam and Capareda, 2015
*Oryza sativa* L.	Asian rice	California	Straw	Yes	Nam and Capareda, 2015
*Phaseolus vulgaris* L.	Common bean	Ecuador	Waste 1	No	Pineda-Insuasti et al., 2014
		Minas Gerais, Brazil	Waste 2	No	Fialho et al., 2019
*Saccharum* spp. L.	Sugarcane	Ghana	Bagasse	Yes	Titiloye et al., 2013
*Zea mays* L.	Corn	Kansas	Leaves	No	Latshaw and Miller, 1924
			Stems	No	Latshaw and Miller, 1924
			Grain	No	Latshaw and Miller, 1924
			Roots	No	Latshaw and Miller, 1924
			Cobs 1	No	Latshaw and Miller, 1924
			Average	No	Latshaw and Miller, 1924
		Ghana	Stalk	Yes	Titiloye et al., 2013
			Cobs 2	Yes	Titiloye et al., 2013
		Iowa, United States	Ground stover	No	Medic et al., 2012
			Stalk shell	No	Medic et al., 2012
			Stalk pith	No	Medic et al., 2012
			Cob shell	No	Medic et al., 2012
			Whole stover	No	Medic et al., 2012

*Data on Phaseolus vulgaris L. waste were reported by Pineda-Insuasti et al. (2014) and Fialho et al. (2019) and will be labeled “Waste 1” and “Waste 2,” respectively, in further discussion. Waste 1 refers to “harvest wastes, the entire plant” (Pineda-Insuasti et al., 2014). Waste 2 refers to “Straw bean: the residue of production of the bean” (Fialho et al., 2019). Similarly, data for Zea mays L. cobs from Latshaw and Miller (1924) and Titiloye et al. (2013) will be labeled “Cobs 1” and “Cobs 2,” respectively. The row “Zea mays L.—average” denotes average composition of the entire plant, based on organ weights reported by Latshaw and Miller (1924).*

Based on the elemental composition of plants, unit carbon formulas were calculated, as described in the *Properties of Live Matter* section. The results are shown in Table 2. It is interesting to compare the empirical formulas in Table 2 with average formulas of some of the major classes of organisms: bacteria (CH_1.__7_O_0.__4_N_0.2_), fungi (CH_1.__7_O_0.__5_N_0.1_), and algae (CH_1.__7_O_0.__5_N_0.1_) (Popovic, 2019). The C:H ratio of the analyzed plants varies from 1.54 to 1.85, with an average of 1.63, which is close to that of the other classes of organisms. However, the analyzed plants have a C:O ratio from 0.71 to 0.90, with an average of 0.80, which is a significantly higher than the C:O ratio of the other classes of organisms. On the other hand, the C:N ratio of the analyzed plants varies from 7 × 10^–4^ to 0.15, with an average of 0.02, which is lower than that of the other classes of organisms. The great span of C:N ratios in plants originates from its dependence on growth conditions, including soil, meteorological factors, agricultural practices, and application of nitrogen fertilizers, as well as availability of other nutrients involved in N accumulation and cycling in crops. Thus, the average oxidation state of carbon in the analyzed plants is 0.00 (from –0.24 to 0.33), which is slightly higher than the averages for bacteria (–0.3), fungi (–0.4), and algae (–0.4). Moreover, the oxidation state of carbon in carbohydrates is also 0.00, which is in accordance with the fact that carbohydrates are a major constituent of plants.

**TABLE 2:** Elemental composition of the plants considered in this work.

Plant name	Plant part	n_*H*_	n_*O*_	n_*N*_	n_*P*_	n_*S*_	n_*K*_	n_*Mg*_	n_*Ca*_	n_*Al*_	n_*Si*_	n_*Mn*_	n_*Fe*_	n_*Cl*_	M_*bio*_ (g/C-mol)
*Gossypium hirsutum* L.	Stalk	1.64	0.869	0.018		0.002									27.87
*Oryza sativa* L.	Straw	1.60	0.807	0.0044		0.0025									26.69
*Phaseolus vulgaris* L.	Waste 1	1.81	0.81	0.15											28.80
	Waste 2	1.55	0.904	0.028		0.002									30.56
*Saccharum* spp. L.	Bagasse	1.54	0.8320	0.012										0.00099	27.11
*Zea mays* L.	Leaves	1.69	0.80	0.027	0.0019	0.0022	0.0110	0.0025	0.0034	0.0008	0.0268	0.00023	0.0004	0.0018	29.10
	Stems	1.58	0.74	0.016	0.0008	0.0013	0.0085	0.0018	0.0011	0.0001	0.0040	0.00008	0.0003	0.0017	26.98
	Grain	1.85	0.76	0.041	0.0029	0.0012	0.0029	0.0022	0.0002	0.0002	0.0002	0.00018	0.0002	0.0002	26.86
	Roots	1.61	0.77	0.026	0.0011	0.0022	0.0035	0.0020	0.0043	0.0103	0.0449	0.00034	0.0026	0.0009	28.39
	Cobs 1	1.66	0.75	0.026	0.0008	0.0002	0.0031	0.0012	0.0001	0.0005	0.0124	0.00015	0.0001	0.0009	26.25
	Average	1.71	0.77	0.029	0.0018	0.0015	0.0065	0.0021	0.0016	0.0011	0.0125	0.00018	0.0004	0.0011	27.53
	Stalk	1.56	0.8653	0.030										0.00834	28.16
	Cobs 2	1.66	0.8556	0.018										0.0021	27.73
	Ground stover	1.68	0.8080	0.0088		0.0010									26.79
	Stalk shell	1.56	0.7170	0.0007		0.0008									25.09
	Stalk pith	1.58	0.8248	0.0023		0.0013									26.88
	Cob shell	1.58	0.7389	0.0018		0.0006									25.47
	Whole stover	1.54	0.7144	0.0025		0.0012									25.05

*The elemental composition is reported in the form of unit carbon formulas (UCFs or empirical formulas), with the general form C_*nC*_H_*nH*_O_*nO*_N_*nN*_P_*nP*_S_*nS*_K_*nK*_Mg_*nMg*_Ca_*nCa*_Al_*nAl*_Si_*nSi*_Mn_*nMn*_Fe_*nFe*_Cl_*nCl*_. Since all elements are given per mole of carbon, n_*C*_ = 1. For example, the UCF of the sample Saccharurn spp. bagasse is CH_1.5__4_O_0.832__0_N_0.012_ (Pineda-Insuasti et al., 2014).*

Based on the elemental composition, growth reactions were made and stoichiometric coefficients calculated, as described in the *Properties of Growth and Biosynthesis* section, which are summarized in Table 3 and Supplementary Material 3. Growth of plants is autotrophic, using CO_2_ as a source of carbon, which is reduced using energy from light. Moreover, data in Supplementary Material 3 show that the greatest amount of O_2_ is produced by growth of *Z. mays* grain (1.06 O_2_-mol/C-mol), while the lowest is that of *Phaseolus vulgaris* waste (0.92 O_2_-mol/C-mol), with the average being 1.00 O_2_-mol/C-mol. This is in good agreement with the average oxidation state of carbon of 0.00, since while going from +4 in CO_2_ to 0.00 in biomass, 1 mol of carbon takes four electrons, which is exactly the amount released when 1 mol of O_2_ is produced from H_2_O (half reaction: 2H_2_O → O_2_ + 4H^+^ + 4e^–^).

**TABLE 3:** Formulas giving stoichiometric coefficients for the plant growth reactions.

Substance	Stoichiometric coefficient
CO_2_ (g)	–n_*C*_
H_2_O (l)	–½ (n_*H*_ + n_*K*_ + 2n_*Mg*_ + 2n_*Ca*_ + 3n_*Al*_ + 2n_*Mn*_ + 3n_*Fe*_ – 3n_*N*_ – 3n_*P*_ - 2n_*S*_ – 4n_*Si*_ – n_*Cl*_)
NH_4_^+^ (aq)	–n_*N*_
H_2_PO_4_^–^ (aq)	–n_*P*_
SO_4_^2–^ (aq)	–n_*S*_
K^+^ (aq)	–n_*K*_
Mg^2+^ (aq)	–n_*Mg*_
Ca^2+^ (aq)	–n_*Ca*_
Al3+ (aq)	–n_*Al*_
Si(OH)_4_ (s)	–n_*Si*_
Mn^2+^ (aq)	–n_*Mn*_
Fe^3+^ (aq)	–n_*Fe*_
Cl^–^ (aq)	–n_*Cl*_
O_2_ (g)	–$\frac{1}{4}$ (2n_*O*_ + 3n_*N*_ + n_*Cl*_ – n_*H*_ – 5n_*P*_ – 6n_*S*_ – n_*K*_ – 2n_*Mg*_ – 2n_*Ca*_ – 3n_*Al*_ – 4n_*Si*_ – 2n_*Mn*_ – 3n_*Fe*_ – 4n_*C*_)
H^+^ (aq)	–(n_*P*_ + 2n_*S*_ + n_*Cl*_ – n_*N*_ – n_*K*_ – 2n_*Mg*_ – 2_*C*__*a*_ – 3n_*Al*_ – 2n_*Mn*_ – 3n_*Fe*_)
Bio	+1

*The table gives stoichiometric coefficients for growth reactions (Equation 11), of the form CO_2_ (g) + H_2_O (l) + NH_4_^+^ (aq) + H_2_PO_4_^–^ (aq) + SO_4_^2–^ (aq) + K^+^ (aq) + Mg^2+^ (aq) + Ca^2+^ (aq) + Al^3+^ (aq) + Si(OH)_4_ (s) + Mn^2+^ (aq) + Fe^3+^ (aq) + Cl^–^ (aq) → (Bio) + O_2_ (g) + H^+^ (aq). The product “(Bio)” denotes live matter (C_*nC*_H_*nH*_O_*nO*_N_*nN*_P_*nP*_S_*nS*_K_*nK*_Mg_*nMg*_Ca_*nCa*_Al_*nAl*_Si_*nSi*_Mn_*nMn*_Fe_*nFe*_Cl_*nCl*_). The calculated stoichiometric coefficient values are negative for the reactants and positive for the products.*

### Thermodynamic Properties of Live Matter and Biosynthesis

Based on elemental composition, standard thermodynamic properties of the analyzed plants were determined, as described in the *Properties of Live Matter* section. Table 4 gives standard thermodynamic properties of the analyzed plants and plant organs, including standard enthalpy of formation, standard molar entropy, standard Gibbs energy of formation, and standard molar heat capacity at constant pressure. All the standard enthalpies of formation are negative, implying that the biomass has a lower energy content than its constituent elements, due to partial oxidation of all other elements by oxygen and nitrogen (Erickson et al., 1978; Popovic, 2019). All the standard molar entropies are positive, in accordance with the third law of thermodynamics (Atkins and de Paula, 2011; Atkins et al., 2017). Standard molar entropies, *S*^0^*_*m*_*, per C-mol of live matter are around 37 J/C-mol K, laying between that of graphite, 5.740 J/mol K, and carbon in gaseous state, 158.10 J/mol K (Atkins et al., 2017). This indicates that the mobility of C atoms in live matter is greater than in graphite but lower than in the gaseous state. All the standard Gibbs energies of formation are negative, with the average value being –126 kJ/C-mol. The standard molar heat capacities at constant pressure are around 35 J/C-mol K.

**TABLE 4:** Thermodynamic properties of live matter of the analyzed plants.

Plant name	Plant part	Δ*_*f*_H*^0^ (kJ/C-mol)	*S*_*m*_^0^ (J/C-mol K)	Δ*_*f*_G*^0^ (kJ/C-mol)	*C*_*p,m*_^0^ (J/C-mol K)
*Gossypium hirsutum* L.	Stalk	–158.1 ± 2.8	38.1 ± 7.5	–108.7 ± 5.0	35.3 ± 3.4
*Oryza sativa* L.	Straw	–116 ± 11	36.1 ± 7.1	–69 ± 11	33.9 ± 3.3
*Phaseolus vulgaris* L.	Waste 1	–187 ± 25	41.4 ± 8.1	–133 ± 25	38.3 ± 3.7
	Waste 2	–198 ± 22	37.8 ± 7.4	–149 ± 23	35.3 ± 3.4
*Saccharum* spp. L.	Bagasse	–156.2 ± 2.7	36.1 ± 7.1	–109.5 ± 4.7	34.0 ± 3.3
*Zea mays* L.	Leaves	–211 ± 25	37.8 ± 7.5	–162 ± 25	36.0 ± 3.5
	Stems	–171 ± 24	35.0 ± 6.9	–125 ± 25	33.5 ± 3.2
	Grain	–180 ± 26	39.1 ± 7.7	–129 ± 26	36.1 ± 3.5
	Roots	–227 ± 24	36.4 ± 7.2	–180 ± 25	35.3 ± 3.4
	Cobs 1	–182 ± 25	36.3 ± 7.2	–135 ± 25	34.4 ± 3.3
	Average	–189 ± 25	37.3 ± 7.3	–141 ± 25	35.2 ± 3.4
	Stalk	–158.8 ± 2.8	37.4 ± 7.4	–110.4 ± 4.9	35.0 ± 3.4
	Cobs 2	–159.2 ± 2.8	38.1 ± 7.5	–109.9 ± 4.9	35.3 ± 3.4
	Ground stover	–182 ± 24	37.2 ± 7.3	–133 ± 24.6	34.6 ± 3.3
	Stalk shell	–158 ± 25	33.9 ± 6.7	–114 ± 24.9	32.4 ± 3.1
	Stalk pith	–182 ± 23	36.2 ± 7.1	–135 ± 23.8	34.0 ± 3.3
	Cob shell	–163 ± 24	34.5 ± 6.8	–118 ± 25	32.8 ± 3.1
	Whole stover	–156 ± 25	33.6 ± 6.6	–113 ± 25	32.2 ± 3.1

*The table includes standard enthalpy of formation (Δ_*f*_H^0^), standard molar entropy (S_*m*_^0^), standard Gibbs energy of formation (Δ_*f*_G^0^), and standard heat capacity at constant pressure (C_*p*__,m_^0^) of anhydrous live matter.*

**FIGURE 1:**
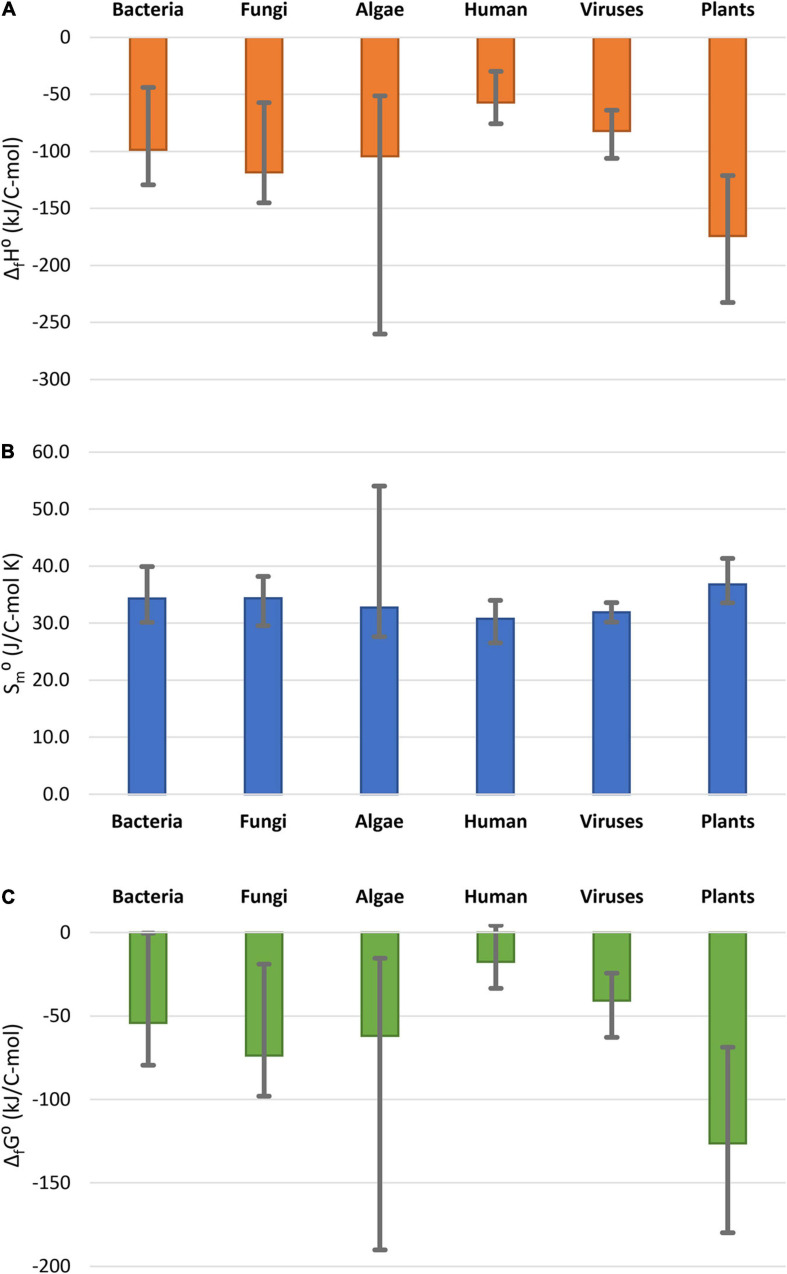
Comparison of thermodynamic properties of formation of live matter: **(A)** standard enthalpy of formation, Δ*_*f*_H*^0^; **(B)** standard molar entropy, *S*_*m*_^0^; **(C)** standard Gibbs energy of formation Δ*_*f*_G*^0^. The columns represent the average thermodynamic parameters for the organism groups, while the error bars show the spread in thermodynamic properties within the groups. Data for bacteria, fungi, and algae taken from Popovic (2019). Data for human tissues taken from Popovic and Minceva (2020c). Data for viruses are taken from (Popovic and Minceva, 2020a,b).

Thermodynamic properties of the analyzed plants are compared with those of other organisms in Figure 1. The data for other classes of organisms are summarized in Supplementary Material 2. Data in Figure 1 represent the average of values for 14 bacteria, 11 fungi, 11 algae, and 18 virus species, as well as 30 human tissues. Figure 1 shows that standard enthalpy and Gibbs energy *of formation* of the analyzed plants is among the most negative measured, but within the span of algae, which are also photosynthetic organisms. Plants have a more negative standard enthalpy and Gibbs energy of formation, indicating a greater energy content and lower oxidation state of carbon in the biomass, compared with other classes of organisms. The reason for this might be that plants gain energy directly from the sun, while heterotrophic organisms have to obtain energy from food. This is supported by the fact that algae, which are also photosynthetic organisms, also have highly negative enthalpy and Gibbs energy of formation. Standard molar entropies of all the organisms in Figure 1 are very similar. Furthermore, plants use a part of the stored energy in catabolic processes to maintain homeostasis, as well as for growth.

Thermodynamic properties of biosynthesis were calculated for the analyzed plants and plant organs, as described in the *Properties of Growth and Biosynthesis* section. The results are given in Table 5. The average standard enthalpy and Gibbs energy of growth are +454.4 and +463.0 kJ/C-mol, respectively, while the average standard entropy of growth is –29.1 kJ/C-mol. To compare, Gibbs energies of biosynthesis of several organisms have been determined for aerobic growth on a mixture of amino acids and glucose: *Escherichia coli* –45.25 kJ/C-mol, *Pseudomonas* (strain C12B) –18.67 kJ/C-mol, *Bacillus subtilis* –31.75 kJ/C-mol, *Cyanobacteria synechocystis* (strain PCC 6803) –13.74 kJ/C-mol, and *Saccharomyces cerevisiae* –15.90 kJ/C-mol (Popovic and Minceva, 2020a). For *S. cerevisiae*, Gibbs energy of biosynthesis was determined for several environments: anaerobic on glucose –11.05 kJ/C-mol, aerobic on glucose –35.76 kJ/C-mol, aerobic on ethanol +28.86 kJ/C-mol, and aerobic on acetic acid +24.10 kJ/C-mol (Battley, 1998). *Chlorella vulgaris* was found to have a Gibbs energy of biosynthesis of +458 kJ/C-mol, during phototrophic growth (Von Stockar et al., 2011). Therefore, Gibbs energy of biosynthesis of heterotrophic organisms is slightly negative or sometimes slightly positive. The reason for this is that heterotrophic organisms use substrates of biological origin, with a Gibbs energy similar to their own live matter. On the other hand, the analyzed plants and *C. vulgaris* are phototrophic organisms and have highly positive Gibbs energies of biosynthesis of around +500 kJ/C-mol. This is due to the high energy requirement for reduction of carbon from CO_2_. However, phototrophic growth is still thermodynamically feasible, due to photosynthesis (Von Stockar, 2013b).

**TABLE 5:** Thermodynamic properties of biosynthesis of the analyzed plants: standard enthalpy (Δ*_*bs*_H*^0^), entropy (Δ*_*bs*_S*^0^), and Gibbs energy (Δ*_*bs*_G*^0^) of biosynthesis.

Plant name	Plant part	Δ*_*bs*_H*^0^ (kJ/C-mol)	Δ*_*bs*_S*^0^ (J/C-mol K)	Δ*_*bs*_G*^0^ (kJ/C-mol)
*Gossypium hirsutum* L.	Stalk	466.1 ± 2.8	–35.0 ± 7.5	476.5 ± 5.0
*Oryza sativa* L.	Straw	506 ± 11	–29.0 ± 7.1	515 ± 11
*Phaseolus vulgaris* L.	Waste 1	421 ± 25	–44.8 ± 8.1	435 ± 25
	Waste 2	409 ± 22	–41.9 ± 7.4	422 ± 23
*Saccharum* spp. L.	Bagasse	453.9 ± 2.7	–34.7 ± 7.1	464.2 ± 4.7
*Zea mays* L.	Leaves	454 ± 25	–23.8 ± 7.5	461 ± 25
	Stems	455 ± 24	–25.3 ± 6.9	462 ± 25
	Grain	474 ± 26	–22.3 ± 7.7	480 ± 26
	Roots	450 ± 24	–16.3 ± 7.2	455 ± 25
	Cobs 1	455 ± 25	–24.3 ± 7.2	462 ± 25
	Average	460 ± 25	–23.2 ± 7.3	467 ± 25
	Stalk	448.5 ± 2.8	–40.0 ± 7.4	460.3 ± 4.9
	Cobs 2	465.6 ± 2.8	–34.4 ± 7.5	475.8 ± 4.9
	Ground stover	450 ± 24	–27.9 ± 7.3	458 ± 25
	Stalk shell	459 ± 25	–22.5 ± 6.7	466 ± 25
	Stalk pith	437 ± 23	–31.0 ± 7.1	447 ± 24
	Cob shell	456 ± 24	–24.1 ± 6.8	463 ± 25
	Whole stover	457 ± 25	–23.2 ± 6.6	464 ± 25

*Biosynthesis represents a chemical process in which CO_2_, H_2_O, and nutrients are converted into new live matter. Thermodynamic properties of biosynthesis do not include the contribution of photosynthetic energy. The reported thermodynamic properties are for biosynthesis reaction (Equation 11) with stoichiometric coefficients calculated using Tables 2, 3.*

Standard Gibbs energy of formation is by definition the Gibbs energy change of reaction (17), the hypothetic formation of live matter from elements in their standard states (Atkins and de Paula, 2011). The elements represent merely a reference state, relative to which Gibbs energy is measured (Atkins and de Paula, 2011). This reference state was chosen because it is not possible to determine absolute Gibbs energy. On the other hand, Gibbs energy of biosynthesis is the Gibbs energy change of reaction (11), representing formation of live matter from nutrients in its environment (Von Stockar, 2013b). Depending on the nutrients and environment, Gibbs energy of biosynthesis can vary even for a single organism (Battley, 1998).

### Photosynthetic Energy and the Driving Force of Growth

**FIGURE 2:**
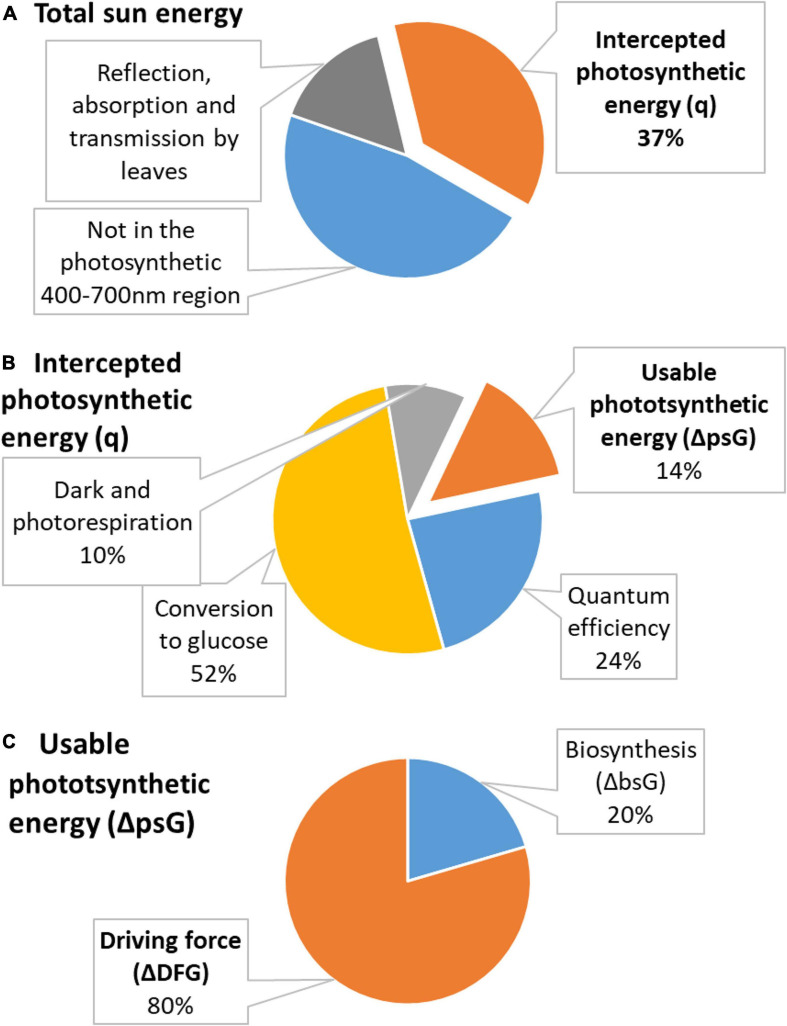
Distribution of energy in photosynthesis. **(A)** Total energy of the sun is partly not in the photosynthetic 400–700 nm region and is partly reflected, absorbed, or transmitted by leaves. The remaining energy is used in photosynthesis and is called intercepted photosynthetic energy (*q*, shown in orange). **(B)** Intercepted photosynthetic energy is not the actual energy used by the plant. A part is lost due to quantum efficiency requirements, in conversion to glucose, and in dark and photorespiration. The remaining energy is usable photosynthetic energy (Δ*_*ps*_G*, shown in orange). **(C)** Usable photosynthetic energy is used partly to provide energy for biosynthesis (Δ*_*bs*_G*) and partly dissipated to make growth occur at a desired rate (Δ*_*DF*_G*).

Energetics of photosynthesis is summarized in Figure 2. The relationship between the biomass accumulation and light energy intercepted by plants is known in agriculture as radiation use efficiency, *RUE*, an important quantifier of crop production, combining photosynthesis and the efficiency of a plant in producing live matter (Monteith and Moss, 1977; Hatfield and Dold, 2019). *RUE* is defined as the slope of the line expressing the relationship between plant biomass, *m*_*bio*_, and intercepted solar radiation

(19) $$m_{bio}=RUE\cdot \sum_{emergence}^{harvest}f_{i}R_{sp}$$

where *f*_*i*_ is the fraction of intercepted photosynthetically active radiation at stage *i* of plant’s development and *R*_*sp*_ is the incoming photosynthetically active radiation (Monteith and Moss, 1977; Sadras et al., 2016; Hatfield and Dold, 2019). Photosynthetically active radiation denotes light in the 400–700 nm range (Murchie et al., 2019). The value of *f*_*i*_ can be found through the equation *f*_*i*_ = *K*_*C*_,*_*i*_* – 0.3, where *K*_*C*_,*_*i*_* is the crop coefficient at stage *i* of plant’s development (Sadras et al., 2016). Crop coefficients are calculated, according to the Food and Agriculture Organization (FAO) method (Doorenbos and Pruitt, 1977), by dividing the crop cycle into four stages: initial, rapid growth, maximum, and declining (Allen et al., 1998; Sadras et al., 2016). For each stage, *K*_*C,i*_ values have been tabulated for many plants (Allen et al., 1998). Similarly, *RUE* has been determined for many plants and depends on environmental conditions, such as availability of nitrogen and other nutrients, temperature, soil water, atmospheric humidity, and wind (Sadras et al., 2016; Hatfield and Dold, 2019; Murchie et al., 2019). More information about *RUE* can be found in Murchie et al. (2019).

*RUE*, defined as the biomass grown per unit light energy, can be used to calculate the intercepted photosynthetic energy, *q*, needed by plants to incorporate 1 mol of carbon into new biomass

(20) $$q=\frac{M_{bio}}{RUE}$$

where *M*_*bio*_ is the molar mass of live matter empirical formula [mass per 1 C-mol, calculated using Equation (2)]. Equation (20) is Equation (19) written for 1 C-mol of plant biomass, with *q* representing the sum term. The values of *q* for the analyzed plants were calculated using literature *RUE* values and are given in Table 6.

**TABLE 6:** Photosynthetic energy and driving force of growth.

Plant name	*RUE* (g/MJ)	*q* (MJ/C-mol)	Δ*_*ps*_G* (MJ/C-mol)	Δ*_*bs*_G* (MJ/C-mol)	Δ*_*DF*_G* (MJ/C-mol)
*Gossypium hirsutum* L.	1.6 ± 1.5	17.0 ± 15.1	–2.5 ± 2.3	+0.476 ± 0.005	–2.1 ± 2.3
*Oryza sativa* L.	2.0 ± 0.4	13.5 ± 3.0	–2.0 ± 0.4	+0.515 ± 0.011	–1.5 ± 0.4
*Phaseolus vulgaris* L.	1.9 ± 0.4	16.0 ± 3.0	–2.4 ± 0.5	+0.428 ± 0.025	–2.0 ± 0.5
*Saccharum* spp. L.	1.4 ± 0.7	19.4 ± 9.5	–2.9 ± 1.4	+0.464 ± 0.005	–2.4 ± 1.4
*Zea mays* L.	2.3 ± 1.9	11.7 ± 9.7	–1.8 ± 1.5	+0.463 ± 0.026	–1.3 ± 1.5

*The table contains radiation use efficiency (RUE), intercepted photosynthetic energy (q), usable photosynthetic energy (Δ_*ps*_G), Gibbs energy of biosynthesis (Δ_*bs*_G), and driving force of growth (Δ_*DF*_G). The Δ_*bs*_G column gives average values for each species from Table 5. RUE data were taken from Kiniry et al. (1989); Sinclair et al. (1992), Kiniry (1994); Muchow et al. (1994), Milroy and Bange (2003); Lindquist et al. (2005), De Silva and De Costa (2012); Hatfield (2014), Zhao et al. (2015); Ghavidel et al. (2016), Huang et al. (2016); Morales-Ruiz et al. (2016), Olivier et al. (2016); Torres et al. (2017), Jia et al. (2018), and Hatfield and Dold (2019).*

It would be interesting to compare the *q*-values from Table 6 with the result of an alternative calculation. Thus, we will estimate *q* in another way. Approximately 5.7 × 10^24^ J of solar energy is irradiated to the Earth’s surface on an annual basis (Miyamoto, 1997). Of this energy, 98–99% is reflected or absorbed by objects, leaving (1.5 ± 0.5)% to be captured by photosynthesis (Thompson et al., 2018). Thus, the total energy absorbed by photosynthetic organisms is (8.6 ± 2.5) × 10^22^ J/year. Photosynthetic organisms utilize this solar energy to fix 2 × 10^11^ tons of CO_2_ per year (Miyamoto, 1997). Since 1 mol of CO_2_ contains 1 mol of carbon and the molar mass of CO_2_ is 44 g/mol, the total amount of carbon fixed by plants is found to be 4.5 × 10^15^ C-mol/year. Therefore, by dividing the total energy (8.6 ± 2.5) × 10^22^ J/year by the amount of carbon fixed 4.5 × 10^15^ C-mol/year, the intercepted photosynthetic energy is found to be *q* = (18.8 ± 6.3) MJ/C-mol. This value is in good agreement with the *q*-values of the analyzed plants from Table 6.

The intercepted photosynthetic energy represents all light energy received by a plant and seems to be very large. However, photosynthesis has several sources of inefficiency: (1) 47% is lost since it is not in the 400–700 nm region used for photosynthesis; (2) 30% is lost in reflection, absorption, and transmission by leaves; (3) 24% for quantum efficiency requirements; (4) 68% in conversion to glucose; and (5) 35–40% on the processes of dark and photorespiration (Sudhakar and Mamat, 2019). Inefficiency sources (1) and (2) are taken into account in Equation (19) by *R*_*sp*_ and *f*_*i*_, respectively. The first source of inefficiency is taken into account when calculating RUE, since Equation (19) contains only *R*_*sp*_ which represents only photosynthetically active 400–700 nm light. Moreover, *f*_*i*_ in Equation (19) takes into account the correction for reflection, absorption, and transmission by leaves. Thus, these two sources of inefficiency are taken into account when calculating *q* using Equation (20), which is derived from Equation (19). However, the remaining three sources of inefficiency have not been taken into account. Thus, the photosynthetic efficiency, *μ*, in converting the intercepted photosynthetic energy *q* into energy usable to plants is

(21) μ=[100%-(24%quantumefficiencyloss)]⋅[100%-(68%conversiontoglucoseloss)]⋅[100%-(40%photorespirationloss)]=15%

Thus, the usable photosynthetic energy, Δ*_*ps*_G*, is the photosynthetic energy actually available for plants to drive their photosynthetic metabolism and is lower than the intercepted photosynthetic energy, *q*

(22) $${△}_{ps}G=-\mu \cdot q$$

The negative sign was added because plants receive energy from the sun as *q* and then spend it on metabolic processes as Δ*_*ps*_G.* A more formal derivation of Equation (22) is given in Supplementary Material 1.

The sum of the usable photosynthetic energy and the Gibbs energy required for biosynthesis, Δ*_*bs*_G*^0^, represents the driving force of growth, Δ*_*DF*_G* (Von Stockar et al., 2011; Von Stockar, 2013b).

(23) $${△}_{DF}G={△}_{ps}G+{△}_{bs}G^{0}$$

The values of *q*, Δ*_*ps*_G*, and Δ*_*DF*_G* for the analyzed plants are given in Table 6. The Δ*_*DF*_G* values greatly vary, even within a species. For example, the driving force for growth of *Saccharum* spp. ranges between –1.0 and –3.8 MJ/C-mol. The reason for this is great variation in reported *RUE*, which is very different for plants grown with irrigation and in dry farming (rain-fed) (De Silva and De Costa, 2012). Moreover, *RUE* of *Z. mays* depends on its growth location (Hatfield and Dold, 2019). However, all the driving force values, despite their differences, have a general trend: they are all negative, making growth of plants a thermodynamically favorable process.

The results for vascular plants obtained in this work can be compared with those for unicellular algae *C. vulgaris*. *C. vulgaris* was found to have an intercepted photosynthetic energy of *q* = 5,000 kJ/C-mol and a Gibbs energy of biosynthesis of Δ*_*bs*_G* = +458 kJ/C-mol (Von Stockar et al., 2011). The average values for the plants analyzed in this research are *q* = 15,537 kJ/C-mol and Δ*_*bs*_G* = +469 kJ/C-mol. To compare the *q-*values, the photosynthetic efficiency, *μ*, has to be taken into account through Equation (22). Thus, usable photosynthetic energy, Δ*_*ps*_G*, is –750 kJ/C-mol for *C. vulgaris* and –2,330 kJ/C-mol for the plants analyzed in this research. These values are then added to the corresponding Δ*_*bs*_G* (Equation 23) to obtain the driving forces of growth, Δ*_*DF*_G*, which are –292 kJ/C-mol for *C. vulgaris* and –1,861 kJ/C-mol for the plants analyzed in this research. The difference between these two values most likely originates from additional processes present in multicellular organisms, such as nutrient uptake by the root (Sondergaard et al., 2004) and great energy used by the root to penetrate through the soil during growth (Colombi et al., 2019; Herrmann and Colombi, 2019). In nutrient limited conditions, plants will spend energy to acquire the limiting nutrient, in accordance with the law of the minimum. Moreover, photosynthesis does not directly drive growth but generates sugars that are oxidized in aerobic respiration to make ATP, which is hydrolyzed to release energy required for plant growth (Hansen et al., 1994, 2004, 2021; Gary et al., 1995; Ellingson et al., 2003). Each step in this process dissipates energy gained during photosynthesis.

The comparison can be carried further by including heterotrophic organisms. Heterotrophic organisms have a driving force of growth of approximately Δ*_*DF*_G* = –500 kJ/C-mol (Heijnen and Van Dijken, 1992; Von Stockar and Liu, 1999; Liu et al., 2007; Von Stockar, 2013b). The corresponding Δ*_*DF*_G* values for photosynthetic growth are –292 kJ/C-mol for *C. vulgaris* and –1,861 kJ/C-mol for the plants analyzed in this research. The value for *C. vulgaris* is on the same order of magnitude as that for heterotrophic organisms. However, the plants analyzed in this research have a significantly greater driving force, which is most likely due to greater energy demands required by multicellular organisms, as discussed above.

Data in Table 6 can be used to compare energetics of C3 and C4 plants. *Saccharum* spp. L. have on average the greatest usable photosynthetic energy Δ*_*ps*_G* and driving force Δ*_*DF*_G*. The reason is that *Saccharum* spp. L. is a C4 plant and is thus more efficient than C3 plants. Representatives of C3 plants, *G. hirsutum* L., and *Phaseolus vulgaris* L. have lower photosynthetic energy Δ*_*ps*_G* and driving force Δ*_*DF*_G*. The lowest values are those of *O. sativa* L., which is also a C3 plant. *Z. mays* L. has the greatest span of Δ*_*ps*_G* and Δ*_*DF*_G*, due to many reported values. Many values have been reported because *Z. mays* is grown throughout the world, under a wide range of conditions. Under optimal conditions, *Z. mays* has a driving force of Δ*_*DF*_G* = –(1.3 + 1.5) = –2.8 MJ/C-mol, which is the one of the highest values for the analyzed plant species. This is in good agreement with it being a C4 plant.

### Driving Force and Growth Rate

The driving force of growth, in the form of negative Gibbs energy, exists to insure that growth occurs at a desired rate. If a process occurs near equilibrium, its Gibbs energy is very close to zero, making it thermodynamically very efficient (Atkins and de Paula, 2011; Atkins et al., 2017). However, processes near equilibrium occur at an infinitely slow rate (Atkins and de Paula, 2011; Demirel, 2014; Atkins et al., 2017), which would not be practical for growth of organisms. Thus, organisms have to make a compromise, between energetic efficiency and growth rate (Von Stockar and Liu, 1999; Von Stockar, 2013c). In other words, they have to waste a certain amount of energy to make growth occur at a desired rate (Von Stockar and Liu, 1999; Von Stockar, 2013c). This wasted energy is the driving force for growth (Von Stockar and Liu, 1999; Von Stockar, 2013c). The driving force, Δ*_*DF*_G*, is hence related to the growth rate of the plant, *r*, through the equation

(24) $$r=-\frac{L}{T}{△}_{DF}G$$

where *L* is a phenomenological coefficient and *T* is temperature (Hellingwerf et al., 1982; Westerhoff et al., 1982; Von Stockar, 2013c; Demirel, 2014; Popovic and Minceva, 2020a,b). The phenomenological coefficient is a proportionality constant that takes into account the kinetic factors (Demirel, 2014) and can be calculated from Equation (24) if the driving force and growth rate are known.

**FIGURE 3:**
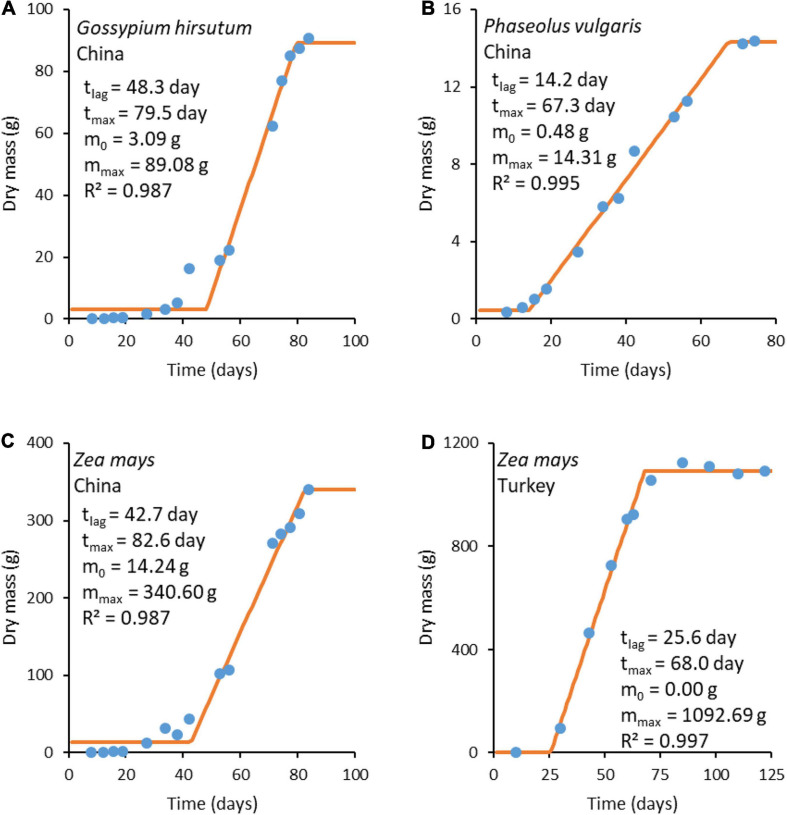
Plant growth curves. The blue circles (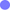) represent experimental data, while the orange lines (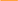) represent a fit made with the three-phase linear model (Equation 25). Experimental data sources: **(A–C)** from Shi et al. (2013) and **(D)** from Koca and Erekul (2016).

Plant growth data were taken from the literature for *G. hirsutum* L. (Shi et al., 2013), *P. vulgaris* L. (Shi et al., 2013), and *Z. mays* L. (Shi et al., 2013; Koca and Erekul, 2016). The growth data were analyzed using the three-phase linear model (Buchanan et al., 1997), which distinguishes three phases in growth: lag, exponential, and stationary (Buchanan et al., 1997; Pérez-Rodríguez and Valero, 2013; Pla et al., 2015). These three phases are analogous to the initial, rapid growth, maximum crop cycle stages, from the FAO method for calculating crop coefficients (Doorenbos and Pruitt, 1977). The original three-phase linear model was modified to describe plant growth

(25a) $$m=m_{0}\;\mathrm{for}\;t\leq t_{lag}$$

(25b) $$m=m_{0}+r\cdot \left(t-t_{lag}\right)\;\mathrm{for}\;t_{lag}<t<t_{max}$$

(25c) $$m=m_{max}\;\mathrm{for}\;t\geq t_{max}$$

(25d) $$r=\frac{m_{max}-m_{0}}{t_{max}-t_{lag}}$$

where *m* is plant mass at time *t*, *m*_0_ is plant mass before the intensive growth phase, *t*_*lag*_ is the duration of the lag phase (time from planting to the intensive growth phase), *r* is the growth rate, and *t*_*max*_ is the moment the steady state is reached and growth ceases. The model has four adjustable parameters: *m*_0_, *m*_*max*_, *t*_*lag*_, and *t*_*max*_. The three-phase linear model is simple, making it a good primary model for obtaining input data for studying more complicated questions, such as the environmental influence on the growth rate (Garthright and Buchanan, 1997). In our case, the three-phase linear model was chosen since it provides a simple and impartial way of determining plant growth rates from growth data. Fitting was made using least squares regression. The fitting results are presented in Figure 3. Growth rate *r* was calculated as the slope of the fitted line using Equation (25d).

The growth rates obtained from fitted functions were combined with Δ*_*DF*_G* (Table 6) to find the phenomenological coefficients using Equation (24). The results are given in Table 7. The obtained phenomenological coefficients vary greatly, depending on plant species and growth conditions. Phenomenological coefficients are specific for every plant species and environmental conditions, including soil, insolation, and water. Thus, the values of the phenomenological coefficients can vary even within one species, if it is cultivated under various conditions. For example, *Z. mays* cultivated in Turkey and China have different phenomenological coefficients, which are influenced by use of different seeds (hybrids) and growth on different soil, as well as temperature, water, and nutrients (Niu and Masabni, 2018).

**TABLE 7:** Growth rates, *r*, and phenomenological *L* coefficients of the analyzed plants.

Plant and growth location	*M*_*bio*_ (g/mol)	*r* (g/day)	*r* (C-mol/day)	*L* (C-mol^2^ K/MJ day)
*Gossypium hirsutum* L., China	27.87	2.75	0.0987	14 ± 16
*Phaseolus vulgaris* L., China	29.68	0.260	0.00877	1.3 ± 0.3
*Zea mays* L., China	26.94	8.17	0.303	70 ± 78
*Zea mays* L., Turkey	26.94	25.76	0.9560	220 ± 250

*Growth rates in g/day were obtained as the slopes of fitted lines shown in Figure 3. The reported growth rates are for the rapid growth stage. The growth rates were converted into C-mol/day, using the equation r(C-mol/day) = r(g/day)/M_*bio*_, where M_*bio*_ is the average molar mass for the plant species (Table 2).*

Data in Table 7 can be interpreted in terms of metabolic processes performed by the analyzed plants. *P. vulgaris* L. has the lowest phenomenological *L* coefficient. The reason is that it is a legume. Legumes can fix nitrogen and thus have a high nitrogen content (*P. vulgaris* L. Waste 1 has the greatest nitrogen content in among all the samples from Table 2). However, nitrogen fixation requires a lot of energy, making less energy available to drive growth (Sinclair and Muchow, 1999). Moreover, by comparing *Z. mays* L. grown in China and Turkey, it can be seen that various sorts of the same species grown under different conditions can have very different phenomenological *L* coefficients and growth rates.

## Conclusion

Empirical formulas with both macro and micro elements were calculated for *Gossypium hirsutum* L. (cotton), *Oryza sativa* L. (Asian rice), *Phaseolus vulgaris* L. (common bean), *Saccharum* spp. L. (sugarcane), and *Zea mays* L. (corn). Moreover, stoichiometry of growth was determined and expressed in the form of growth reactions. Plant live matter and growth process were analyzed by determining their standard thermodynamic properties of formation and biosynthesis, respectively. The average standard Gibbs energy of formation of the analyzed plants was found to be –126 kJ/C-mol. The analyzed plants were found to have among the lowest enthalpies and Gibbs energies of formation relative to the elements, along with algae, compared with bacteria, fungi, viruses, and human tissues. The highly negative enthalpies and Gibbs energies of formation of plants and algae indicate a high energy content, originating from their ability to obtain energy directly from the sun. Standard Gibbs energies of biosynthesis are positive for all the analyzed plants, since photosynthetic growth is based on reducing carbon in CO_2_. The average standard Gibbs energy of biosynthesis of the analyzed plants is +463.0 kJ/C-mol.

Photosynthesis was analyzed through three parameters: intercepted photosynthetic energy, usable photosynthetic energy, and the driving force of growth. Energetics of photosynthesis was analyzed starting from RUE, biomass grown per unit light energy, reported in the literature. The average RUE for the analyzed plants was found to be 1.8 g/MJ. RUE was used to find the intercepted photosynthetic energy, light energy in the photosynthetic 400–700 nm range received by plants. The average intercepted photosynthetic energy was found to be 15.5 MJ/C-mol for the analyzed plants. However, intercepted photosynthetic energy is not completely usable to plants. Thus, it was corrected for photosynthetic efficiency of 15%, to find the usable photosynthetic energy, photosynthetic energy actually available for plants to drive their metabolism. The average usable photosynthetic energy was found to be –2.3 MJ/C-mol for the analyzed plants. The usable photosynthetic energy was added to the standard Gibbs energy of biosynthesis, to find the driving force of growth—energy spent on keeping growth sufficiently far from equilibrium for it to occur at a desired rate. The average driving force of growth of the analyzed plants was found to be –1.9 MJ/C-mol. This value is greater than that of heterotrophic and phototrophic unicellular organisms reported in the literature, most likely due to additional energy requirements by multicellular organisms. Driving forces of growth of C3 and C4 plants were compared. It was found that C4 plants have a greater driving force of growth than C3 plants, which reflects the greater efficiency of C4 photosynthesis.

The driving force of growth is related to the growth rate, the proportionality constant being phenomenological *L* coefficients. Phenomenological *L* coefficients were determined for the first time for the analyzed plants. The phenomenological *L* coefficients of the analyzed plants span two orders of magnitude, depending on plant species and growth conditions. The *L* coefficient of *P. vulgaris* was found to be lower than that of other plants, due to additional energy requirements of nitrogen fixation.

## Data Availability Statement

The original contributions presented in the study are included in the article/Supplementary Material, further inquiries can be directed to the corresponding author/s.

## Author Contributions

MP conceived and designed the research, collected the data, analyzed and interpreted the data, and wrote the manuscript. MM analyzed and interpreted the data, and wrote the manuscript. Both authors contributed to the article and approved the submitted version.

## Conflict of Interest

The authors declare that the research was conducted in the absence of any commercial or financial relationships that could be construed as a potential conflict of interest.
