# Supplementary material for: Standard Thermodynamic Properties, Biosynthesis Rates, and the Driving Force of Growth of Five Agricultural Plants
Source: Front Plant Sci. 2021 May 31;12:671868. doi: 10.3389/fpls.2021.671868 (PMC8202407; doi:10.3389/fpls.2021.671868)
Supplement: Supplementary file 1 [file Table_1.docx]

**Supplementary Material 1: Gibbs energy of photosynthesis**

All electromagnetic radiation is classified as radiation heat transfer (Balmer, 2011). Thus, the incoming solar radiation is essentially heat received by a plant from the sun. Hence, the symbol *q* was used, which is used to denote heat in thermodynamics. Moreover, photosynthesis occurs at constant pressure and heat exchanged at constant pressure corresponds to enthalpy change (Atkins & de Paula, 2011; Balmer, 2011). Thus, the incoming photosynthetic energy *q* leads to an equal change in enthalpy of the system, which will be named Δ*_PAR_H*, the enthalpy change of the plant due to photosynthetically active radiation.

$q=\Delta_{PAR}H$ (B1.1)

Since the plant receives energy from light, both *q* and Δ*_PAR_H* are positive by convention. However, neither *q* nor Δ*_PAR_H* are the actual energy available to the plant, but the Gibbs (free) energy of photosynthetically active radiation, Δ*_PAR_G*

$\Delta_{PAR}G=\Delta_{PAR}H-T\cdot\Delta_{PAR}S$ (B1.2)

where *T* is temperature and Δ*_PAR_S* is the entropy change due to photosynthetically active radiation (Atkins & de Paula, 2011). In other words, according to the second law of thermodynamics, no process is perfectly efficient, including photosynthesis. Δ*_PAR_H* represents all the energy received by the plant from light. But, a part of this energy is lost, which is quantified by *T*∙Δ*_PAR_S*. The remaining energy, Δ*_PAR_G*, is available to the plant for driving its metabolism. A simpler relationship between Δ*_PAR_H* and Δ*_PAR_G* can be made using photosynthetic efficiency *µ*.

$\Delta_{PAR}G=\mu\cdot\Delta_{PAR}H$ (B1.3)

The value of *µ* is 15%, according to equation (21). The photosynthetic efficiency *µ* takes into account all the losses due to inefficiency in photosynthesis, discussed in Section 3.3.

So far, the plant has gained Δ*_PAR_G* of usable energy from light. Now it is time for it to be spent. Δ*_PAR_G* forms the energetic budget of the plant, which is to be spent. Thus, it is equal in magnitude to the energy that will be spent, but opposite in sign.

$\Delta_{ps}G=-\Delta_{PAR}G$ (B1.4)

where Δ*_ps_G* represents the photosynthetic energy available for the plant to spend, or usable photosynthetic energy. Combining equations (B1.4), (B1.3) and (B1.1) gives equation (22) from Section 3.3.

$\Delta_{ps}G=-\mu\cdot\Delta_{PAR}H=-\mu\cdot q$ (B1.5)

The available photosynthetic energy Δ*_ps_G* is then used for two purposes. First to provide energy for biosynthesis, which is quantified by Δ*_bs_G* and to ensure that growth occurs at a desired rate Δ*_DF_G*.

$\Delta_{DF}G=\Delta_{ps}G+\Delta_{bs}G$ (B1.6)

Δ*_bs_G* is added to Δ*_ps_G*, rather than subtracted, since Δ*_bs_G* is positive, while Δ*_ps_G* is negative. Thus, their absolute values are subtracted from each other. In absolute values: |Δ*_ps_G*| = |Δ*_bs_G*| + |Δ*_DF_G*|.
