# Supplementary material for: Standard Thermodynamic Properties, Biosynthesis Rates, and the Driving Force of Growth of Five Agricultural Plants
Source: Front Plant Sci. 2021 May 31;12:671868. doi: 10.3389/fpls.2021.671868 (PMC8202407; doi:10.3389/fpls.2021.671868)
Supplement: Supplementary file 2 [file Table_2.docx]

**Supplementary Material 2: Thermodynamic properties of organisms**

Thermodynamic properties of some classes of organisms: standard enthalpy of formation (Δ*_f_H⁰*), standard molar entropy (*S_m_⁰*) and standard Gibbs energy of formation (Δ*_f_G⁰*), of anhydrous live matter. The values in the table represent averages for 14 bacteria, 11 fungi, 11 algae and 18 virus species, as well as 30 human tissues. The data is in *X*±Δ*X* format, where *X* represents the average value, while Δ*X* is the maximum deviation from the average within the group.

| **Name** | **Δ_f_H⁰ (kJ/mol)** | | |  | **S_m_⁰ (J/mol K)** | | |  | **Δ_f_G⁰ (kJ/mol)** | | |  | **References** |
| --- | --- | --- | --- | --- | --- | --- | --- | --- | --- | --- | --- | --- | --- |
| Bacteria | -99 | ± | 55 |  | 34.3 | ± | 5.6 |  | -54 | ± | 54 |  | (Popovic, 2019) |
| Fungi | -118 | ± | 61 |  | 34.3 | ± | 4.8 |  | -74 | ± | 55 |  | (Popovic, 2019) |
| Algae | -104 | ± | 156 |  | 32.7 | ± | 21.3 |  | -62 | ± | 128 |  | (Popovic, 2019) |
| Human | -57 | ± | 27 |  | 30.8 | ± | 4.2 |  | -18 | ± | 22 |  | (Popovic and Minceva, 2020c) |
| Viruses | -82 | ± | 24 |  | 31.9 | ± | 1.7 |  | -41 | ± | 22 |  | (Popovic and Minceva, 2020a, 2020b) |
